# Support, Monitoring, and Reminder Technology for Mild Dementia (SMART4MD) for People With Mild Cognitive Impairment and Their Informal Caregivers: Cost-Effectiveness Analysis

**DOI:** 10.2196/77808

**Published:** 2026-05-22

**Authors:** Zartashia Ghani, Johan Jarl, Peter Anderberg, Johan Sanmartin Berglund, Maria Quintana Aparicio, Pilar Barnestein-Fonseca, Selim Cellek, Fermín Mayoral-Cleries, Maite Garolera, Gloria Guerrero-Pertiñez, Karen Hayden, Carmel Moore, Jufen Zhang, Dominic Trepel, Sanjib Saha

**Affiliations:** 1 Blekinge Institute of Technology Karlskrona Sweden; 2 Department of Clinical Science (Malmö) Health Economics Unit Lund, Skane Sweden; 3 Consorci Sanitari de Terrassa Torrebonica Spain; 4 Instituto CUDECA de Estudios e Investigación en Cuidados Paliativos, Fundación CUDECA Málaga Spain; 5 Anglia Ruskin University Chelmsford United Kingdom; 6 Mental Health Clinical Management Unit Regional University Hospital of Malaga Malaga Spain; 7 Consorci Sanitari de Terrassa Barcelona Spain; 8 Trinity College Dublin Dublin Ireland

**Keywords:** cost-effectiveness, mHealth, dementia, mild cognitive impairment, Informal Caregivers

## Abstract

**Background:**

Support, Monitoring, and Reminder Technology for Mild Dementia (SMART4MD), a customized tablet app, was developed to improve or maintain the quality of life of people with mild cognitive impairment (MCI) and their informal caregivers.

**Objective:**

This study conducts an 18-month economic evaluation of the SMART4MD app, in addition to standard care, compared with standard care alone in Sweden and Spain, from a health care provider perspective.

**Methods:**

In a pragmatic randomized controlled trial, people with MCI and their informal caregivers were randomized to the intervention and control groups. Health care costs, quality-adjusted life-years (QALYs), and incremental cost-effectiveness ratios (ICERs) were measured in 345 Swedish people with MCI and their informal caregivers, and in 347 Spanish people with MCI.

**Results:**

The analysis showed higher incremental costs and lower QALYs for Swedish people with MCI than for controls, whereas higher incremental costs and higher QALYs were observed for Spanish people with MCI. The intervention was not found to be cost-effective for Swedish informal caregivers, with an ICER of €78,000/QALY (€1=US $1.16).

**Conclusions:**

The differing findings regarding cost-effectiveness for people with MCI in Sweden and Spain highlight the need for further research with extended follow-up, ideally involving a larger sample size and conducted across different national contexts.

**Trial Registration:**

ClinicalTrials.gov NCT03325699; https://clinicaltrials.gov/ct2/show/NCT03325699

## Introduction

Memory loss, or a decline in cognitive abilities such as language and spatial skills, is an early sign of diminishing mental abilities, referred to as mild cognitive impairment (MCI). MCI is a condition characterized by cognitive decline that exceeds what is typical for a person’s age and education level, yet does not significantly impact daily activities [1]. This condition has the potential to progress into dementia over time. Approximately half of the people with MCI develop dementia within 3 years [2]. Dementia imposes a substantial financial burden on the health care sector, as well as emotional and financial stress on close relatives. The annual incidence of dementia in Sweden is estimated at 25,000 cases, with a corresponding societal cost of €45,000 (€1=US $1.16) per person [3]. The age-adjusted incidence rate of dementia among individuals aged 65 years or older in Spain is reported to be 6.66 per 1000 persons [4]. The total societal cost of dementia in Spain was reported to be €8268.5 million in 2018 [5]. According to the Dementia in Europe Yearbook 2019 [6], the prevalence of dementia in Sweden and Spain by 2050 is projected to be approximately 2.63% and 3.99% of the total population, respectively. Research further demonstrates that people with dementia incur higher health care costs up to 10 years before diagnosis compared with those without dementia [7].

MCI poses challenges to activities of daily living. People with MCI need active assistance from informal caregivers, for example, with taking medication, keeping medical appointments, and managing other daily activities. Caregiving responsibilities increase as the cognitive abilities of people with MCI decline over time [8]. This is expected to lead to emotional and mental stress among informal caregivers and affect the quality of life (QoL) of both people with MCI and their informal caregivers [9].

Smartphone/tablet apps have been suggested as useful tools to assist people with MCI in performing activities of daily living [10]. Regular use of smartphone/tablet apps by people with MCI may help them develop routines that become even more important, and thus beneficial, with increasing cognitive decline. Maintaining autonomy is expected to ease the burden on informal caregivers and improve the well-being of both people with MCI and their informal caregivers. Based on this notion, the tablet-based app Support, Monitoring, and Reminder Technology for Mild Dementia (SMART4MD) was developed to improve or maintain the QoL of people with MCI and their informal caregivers [11]. A pragmatic randomized controlled trial (RCT) was conducted in 3 European countries (Sweden, Spain, and Belgium) to study the effectiveness of the app in comparison with standard care over an 18-month period, from December 2017 to September 2020 (ClinicalTrials.gov registration number NCT03325699).

Our findings regarding the efficacy of the SMART4MD app over the 18-month trial period indicated a significant improvement in the quality of life at 6-month (measured as quality-adjusted life-years [QALYs]) of informal caregivers and dyads in the intervention group (authors’ unpublished observations). Economic evaluations of such interventions can assist health policy makers and reimbursement agencies in making informed and transparent decisions. A short-term economic evaluation of the SMART4MD intervention, based on 6-month trial data, has previously been published. The results indicate that the intervention might yield benefits for informal caregivers in terms of reducing caregiving burden and improving their QoL [12]. However, the short follow-up period is not expected to fully capture the effects on health and QoL. Therefore, the purpose of this study is to assess the 18-month cost-effectiveness of the SMART4MD tablet-based intervention, in addition to standard care, compared with standard care, from the perspective of the health care provider in the Swedish and Spanish settings.

## Methods

### Study Design and Reporting Standards

This economic evaluation was conducted following the Consolidated Health Economic Evaluation Reporting Standards (CHEERS) guidelines (see Multimedia Appendix 1), using data from the SMART4MD trial [13].

### The SMART4MD Trial

SMART4MD aims to improve or maintain the quality of life of people with MCI and their informal caregivers. SMART4MD is a customized mobile health (mHealth) app for tablets, adapted for people with MCI. The app was developed by Healthbit Ltd [14] in consultation with people with MCI, their informal caregivers, and health care professionals. The app’s primary features include medication and health care appointment reminders, cognitive support activities such as games and photos, and a health information feature that allows users to share their daily health status or health-related issues with others. The idea was to support people with MCI while also helping establish a routine in their daily lives that could be maintained as the disease progressed.

The SMART4MD trial was conducted at 4 sites in Sweden, Spain, and Belgium: (1) Blekinge Institute of Technology (BTH), Karlskrona, Sweden; (2) Consorci Sanitaria de Terrassa (CST), Barcelona, Spain; (3) Servicio Andaluz de Salud, Málaga, Spain; and (4) University College Leuven-Limburg, Belgium. One of the Spanish sites (Servicio Andaluz de Salud) was excluded from the analysis due to the unavailability of cost data, whereas the Belgian site was excluded due to insufficient participants (5 dyads) and the unavailability of both cost and effect data. However, we performed a scenario analysis for people with MCI from 1 Spanish site (CST), where inpatient and outpatient cost data were available only for people with MCI. Thus, this study conducted the primary analysis using complete 18-month trial data for both people with MCI and informal caregivers from the Swedish site (BTH), and a scenario analysis only for people with MCI from the Spanish site (CST).

The recruitment of participants was carried out through primary care and secondary care services (memory clinics), as well as outpatient clinics, day hospitals, specialist mental health care units, geriatric medicine units, and neurology service units. Older adults (≥55 years) who could administer their own medication and had a Mini-Mental State Examination (MMSE) score ranging from 20 to 28 were eligible for inclusion. Further inclusion criteria were that participants were physically fit to use the app, had an informal caregiver, and had experienced impaired cognition for more than 6 months. Older adults were excluded if they scored above 11 on the 15-item Geriatric Depression Scale, indicating severe depression [15], or if they had a life expectancy of less than 3 years due to a life-limiting illness. A research nurse used information collected at the baseline visit and from participants’ medical records to assess life expectancy.

A total of 345 dyads (people with MCI and their main informal caregiver) were randomized to either the intervention or control group, resulting in 173 dyads in the intervention group and 172 dyads in the control group. In addition, 347 people with MCI (intervention group, n=174; control group, n=173) participated from the Spanish site (CST). Participants randomly assigned to the intervention group received the SMART4MD app on data-enabled tablets, along with standard care. The intervention group also received a 1.5-hour training session, led by a research nurse, to demonstrate the functions of the app and was instructed to use the app daily, with assistance from their informal caregiver if needed. As data-enabled tablets and training were provided to trial participants, no technical prerequisites regarding familiarity with, or ownership of, smartphones or tablets were required for participation.

Participants in the control group received only standard care. Standard care in Sweden includes an annual in-person or telephonic visit to a physician for routine checkups and the renewal of drug prescriptions, as well as necessary laboratory tests, if required. Standard care in Spain includes a visit to a health center, where patients receive diagnostic, therapeutic, and preventive services and are potentially referred to specialists if necessary [16]. As all dyads continued to seek health care as needed, the extent of health care contacts varied between participants due to differences in general health, comorbidities, and health-seeking behavior. Further information on the SMART4MD app and the trial is described in detail elsewhere [11].

### Ethics Considerations

The study was conducted in accordance with the ethical principles of the Declaration of Helsinki (World Medical Association, 2013). Ethical approval for the SMART4MD trial was obtained from the Regional Ethical Review Board in Sweden (LU numbers 650-00 and 744-00) and the local research ethics committee in Spain (approval number 02-16-107-029). Before participation, all older adults received comprehensive oral and written information about the study, including its objectives, data protection measures, and the voluntary nature of participation. Written informed consent was obtained from each participant before enrollment and reaffirmed at each data collection point.

### Resource Use Measurement and Estimating Costs

A health care provider’s (regional council’s) perspective was adopted; thus, only costs incurred by the health care provider were included. Costs related to community, social, and home support services were not included, as these costs fall on municipalities rather than the health care provider. All costs were estimated in Swedish kronor (SEK) and reported in 2020 euros (€), using an exchange rate of 10.4867 SEK/€ [17].

All types of health care utilization for both inpatient and outpatient care were included in the study. Outpatient care includes both primary care and specialized outpatient clinics. Costs arising from the intervention include the purchasing cost of tablets, mobile data, and the time of the research nurse who provided training on how to use the app. All managerial costs related to the development and execution of the RCT were classified as research costs and were therefore excluded from the analysis.

Health care utilization and costs were collected from the Blekinge regional council health care registers [18], where costs were assigned to each health care episode using diagnosis-related groups [19]. In cases of missing outpatient health care costs, the average costs of visits in primary care and in each specialized outpatient clinic, stratified by type of health care personnel, were used (Table S1 in Multimedia Appendix 2). In a few cases, both cost and type of health care personnel were missing; in these instances, the average cost for each type of visit in specialized clinics was used. In cases where inpatient care costs were missing, the average daily cost was used.

### Measurement of Effectiveness

#### EQ-5D-3L Index Score

The EQ-5D-3L questionnaire is a preference-based instrument that includes 5 attributes: mobility, self-care, usual activities, pain/discomfort, and anxiety/depression. Each attribute can be rated at 3 levels: no problem, some problem, and extreme problem, creating a total of 243 possible health states [20]. These health states are translated into a score ranging from 0 (equivalent to dead) to 1 (perfect health), based on population preferences [21]. The EQ-5D-3L questionnaire was collected for both people with MCI and informal caregivers at baseline and at 3 follow-up points (6, 12, and 18 months). EQ-5D-3L utilities [20] were estimated using the Swedish experience-based tariff [22].

#### Quality-Adjusted Life-Years

QALYs were included as the primary outcome measure for both people with MCI and informal caregivers, based on the EQ-5D-3L index score. The change in QALYs was estimated using the area-under-the-curve approach [23]. QALYs were calculated for each follow-up assessment at 6, 12, and 18 months. A 180-day window, with a tolerance of +18 or –18 days, was designated for each visit in the SMART4MD trial. However, as is common in trials, some participants’ visits occurred outside this 180-day period. Consequently, QALYs were calculated using the actual number of days between visits instead of the fixed 180-day intervals. To determine the total QALYs for the entire 18-month trial period, the QALYs from each follow-up assessment were summed, and participants who missed any follow-up assessments were excluded.

#### Quality of Life in Alzheimer’s Disease Scale

Quality of Life in Alzheimer’s Disease Scale (QoL-AD) measures disease-specific quality of life and comprises 13 items covering interpersonal, environmental, functional, physical, and psychological aspects of people with dementia [24]. Scores of 1 (poor), 2 (fair), 3 (good), and 4 (excellent) were combined to yield a total score, with higher totals indicating better quality of life [25]. We employed both self-rated and proxy-rated versions of QoL-AD. People with MCI were interviewed to obtain QoL-AD scores, while informal caregivers completed a self-administered questionnaire. Following Logsdon et al [26], we combined the 2 ratings into a weighted composite QoL-AD score, in which the rating of people with MCI was given 2 times the weight of the informal caregiver’s proxy rating.

#### Mini-Mental State Examination

The MMSE score measures cognitive functioning [27]. It is a standardized assessment tool used to evaluate the cognitive abilities of people with MCI. This test focuses on memory, attention, orientation, and language skills, with a maximum score of 30 [27]. A higher MMSE score suggests a more positive outcome, while a score below 24 indicates the presence of dementia. However, there is no agreed-upon cutoff score for the MMSE to define MCI in the scientific literature [28].

#### Short-Form 12-Item Zarit Burden Interview

Caregiving burden was included as a secondary outcome measure for informal caregivers and was estimated using the short-form 12-item Zarit Burden Interview (ZBI-12). This self-reported instrument contains 12 items measuring the effect of caregiving activities on the informal caregiver’s leisure time and overall health. A 5-point Likert scale (0=never to 5=nearly always) was used to score each question. The maximum score of the ZBI-12 is 48 points, where a score of less than 17 represents no burden [29]. To align with other outcome measures, we inverted the ZBI-12 score so that a higher score indicates a more favorable outcome. Thus, a score equal to or above 31 represents no burden.

### Analysis of Cost-Effectiveness

The results are presented as an incremental cost-effectiveness ratio (ICER), which is the ratio of between-group differences in costs and effects, measured from baseline to the end of follow-up (18 months). According to the Swedish National Board of Health and Welfare, an ICER above 500,000 SEK (€48,876) is considered a high cost per QALY gained and is therefore used as the willingness-to-pay (WTP) threshold in this study [30]. Thus, if the estimated cost per QALY was below this threshold, the use of the SMART4MD app was considered cost-effective from a health care provider’s perspective, compared with standard care. We also expressed the results in terms of net monetary benefit (NMB), which is calculated as follows: (incremental benefit × WTP) − incremental cost. The NMB provides results in monetary terms with a known threshold value of benefit. A positive NMB indicates that the tablet-based app is cost-effective compared with standard care. Some advantages of calculating NMB are that it is linear, has a simple sampling distribution, and is more stable than the ICER in cases of marginal differences in effectiveness between the intervention and comparator [31]. As both Swedish and Spanish guidelines recommend using a 3% discount rate in economic evaluations, we applied this rate to discount both QALYs and costs for the last 6 months of the trial [32].

The uncertainty around the cost-effectiveness results was explored using 5000 bootstrap resamples of the ICER, presented graphically on a 4-quadrant cost-effectiveness (CE) plane [33]. A cost-effectiveness acceptability curve (CEAC) was also derived to present the probability of the intervention being cost-effective at different WTP thresholds.

### Sensitivity and Subgroup Analyses

We conducted several sensitivity and subgroup analyses to assess the uncertainty around the base-case results. Sensitivity analyses were reported for (1) people with MCI, (2) caregivers, and (3) dyads, where applicable (Textbox 1).

**Additional analyses.**

**1.Multiple imputation for missing quality-adjusted life-years**

Missing information on the EQ-5D-3L index score was imputed using multiple imputation (20 datasets), based on baseline age, sex, education, living arrangements, EQ-5D-3L index score, Quality of Life in Alzheimer’s Disease Scale score of people with mild cognitive impairment, Mini-Mental State Examination score, 12-item Zarit Burden Interview score, and intervention/control group [34]. After generating the multiple datasets, pooled estimates were computed [35].

**2. Intervention cost**

The intervention costs include the tablet purchasing cost, mobile data, and the time of the research nurse conducting training sessions on app usage. As these costs are not expected to be covered by the health care provider in the case of widespread implementation, they were included only in a sensitivity analysis.

**3. Removing zero health care cost**

During the trial, people with mild cognitive impairment were expected to have health care contacts due to their age and health condition. Thus, dyads with zero health care costs were removed in a sensitivity analysis, as these could be considered outliers.

**4. Subgroup analysis—sex**

Results were stratified by sex to explore whether they were influenced by differences in health-seeking behavior between men and women.

**5. Subgroup analysis—age**

Results were stratified by age (up to 70 vs ≥70 years) to explore whether they were influenced by differences in familiarity with and use of tablets, which is assumed to be correlated with age [36,37].

**6. Subgroup analysis—Mini-Mental State Examination**

Results were stratified based on baseline Mini-Mental State Examination score (≤26 vs >26) [38] to explore whether they were influenced by the level of cognitive decline at the time of introduction to the app. Furthermore, prior research indicates that this cutoff is advantageous due to its enhanced specificity and sensitivity [38,39]. Moreover, this threshold aligns with the median of our sample.

**7. Subgroup analysis—Zarit burden**

Results were stratified based on baseline Zarit burden score (no burden [12-item Zarit Burden Interview >31] vs some burden [12-item Zarit Burden Interview ≤31]) to explore whether they were influenced by the level of caregiving burden.

**8. User behavior**

Usage of the Support, Monitoring, and Reminder Technology for Mild Dementia (SMART4MD) app was assessed by the number of times it was launched during the first 12-month period (information was unavailable for the last 6 months of the trial). Results were stratified based on high versus low usage (median cutoff of 125 launches in our sample during the first year) to explore whether more frequent use of the app influenced the results.

### Scenario Analyses

Two scenario analyses were performed. The first scenario analysis was conducted for one of the Spanish sites (CST), where cost data for people with MCI were available. A Spanish tariff was used to calculate QALYs [40]. Although Spain does not use an explicit threshold to establish the cost-effectiveness of health care interventions, a threshold of €30,000 per QALY is the most frequently cited in Spanish economic evaluation studies [41]. Therefore, we also used €30,000 per QALY as the threshold to establish the cost-effectiveness of the SMART4MD app for people with MCI participating at the Spanish site in this study. Health care utilization data were collected from Hospital de Terrassa, Terrassa Sanitary Consortium, Terrassa (Barcelona). All costs were estimated in euros (€) and reported in 2019 prices [42].

The second scenario analysis was conducted to evaluate the cost-effectiveness of people with MCI who participated from both BTH and CST sites, utilizing costs, QALYs, QoL-AD, and MMSE scores. The European EQ-5D VAS tariff was used to calculate QALYs in the second scenario [43], and a threshold value of €30,000 per QALY was applied [41].

### Statistical Analysis

Intergroup (between intervention and control groups) statistical differences were assessed using an independent *t* test (unpaired and 2-tailed) for continuous variables and a chi-square test for categorical variables. Intragroup (within intervention and control groups) statistical differences were assessed using a paired *t* test. The scores of both people with MCI and their informal caregivers were combined to calculate dyad scores, and the results were presented as mean (SD) or mean differences. All analyses were conducted using Stata/SE 15.1 (StataCorp LP).

## Results

### Baseline Characteristics and Follow-Up Outcomes

Table 1 presents baseline and 18-month follow-up demographic characteristics of people with MCI and informal caregivers, where no considerable differences were observed between the intervention and control groups at baseline. At the 18-month follow-up, both people with MCI and dyads had significantly lower QALYs (*P*=.047 and .03, respectively) in the intervention group compared with the control group at the 12-month follow-up, whereas informal caregivers (*P*=.02) and dyads (*P*=.03) had significantly higher EQ-5D-3L index scores in the intervention group compared with the control group at the 18-month follow-up (Table S2 in Multimedia Appendix 2).

After 18 months, of the 345 dyads, 73 (21.2%) had left the trial, with the most prominent reason being “simply do not want to participate” (45/73, 62%), followed by physical (9/73, 12%) and cognitive reasons (6/73, 8%; see Figure S1 in Multimedia Appendix 2). Further, we excluded 5 informal caregivers who had not reported any health outcomes. Three people with MCI had changed their primary caregivers during the trial. As it is not reasonable to compare different caregivers in terms of health outcomes, these cases were excluded from the analyses (see Figure S1 in Multimedia Appendix 2).

The dropout versus nondropout analysis of baseline characteristics for people with MCI showed that those who dropped out had significantly worse EQ-5D-3L index score at baseline in both the intervention (*P*=.03) and control (*P*=.0097) groups. People with MCI who dropped out of the control group were more likely to live with a spouse or partner and had worse MMSE scores. The dropout analysis for informal caregivers in the control group showed that, at baseline, those who dropped out had higher Zarit burden scores and reported worse proxy QoL-AD scores on behalf of people with MCI (see Tables S3 and S4 in Multimedia Appendix 2).

**Table 1:** Demographic characteristics of people with MCI^a^ and informal caregivers at baseline and 18-month follow-up.

People with MCI																		Informal caregiver																		
			Baseline						18-month follow-up							Baseline											18-month follow-up									
			Intervention (n=173)		Control (n=172)		*P* value		Intervention (n=131)		Control (n=141)		*P* value		Intervention (n=173)					Control (n=172)			*P* value			Intervention (n=127)			Control (n=137)			*P* value				
Age (years), mean (SD)			76 (5.06)		76 (5.18)		.74		77 (5.10)		78 (5.07)		.79		70 (10.45)					69 (11.35)			.51			71 (10.39)			71 (11.22)			.93				
**Gender, n (%)**							.47						.54										.67									.69				
	Male	97 (56.1)		103 (59.9)				76 (58.0)		87 (61.7)				57 (32.9)					53 (30.8)						39 (30.7)			39 (28.5)								
	Female	76 (43.9)		69 (40.1)				55 (42.0)		54 (38.3)				116 (67.1)					119 (69.2)						88 (69.3)			98 (71.5)								
**Education^b^** **, n (%)**							.07						.01										.37									.30				
	Elementary education	57 (32.9)		64 (37.2)				47 (35.9)		52 (36.9)				44 (25.4)					36 (20.9)						38 (29.9)			31 (22.6)								
	Secondary education	57 (32.9)		38 (22.1)				44 (33.6)		27 (19.1)				64 (37.0)					60 (34.9)						45 (35.4)			50 (36.5)								
	Higher education	58 (33.5)		70 (40.7)				39 (29.8)		62 (44.0)				63 (36.4)					75 (43.6)						42 (33.1)			56 (40.9)								
**Civil status, n (%)**							.64						.52										.64									.67				
	Single	46 (26.6)		42 (24.4)				38 (29.0)		36 (25.5)				23 (13.3)					20 (11.6)						19 (15.0)			18 (13.1)								
	Married/living together	127 (73.41)		130 (75.6)				93 (71.0)		105 (74.5)				150 (86.7)					152 (88.4)						108 (85.0)			119 (86.9)								
**Living arrangements, n (%)**							.57						.01										.50									.29				
	Single	41 (23.7)		42 (24.4)				35 (26.7)		25 (17.7)				18 (10.4)					18 (10.5)						13 (10.2)			7 (5.1)								
	Spouse/common law partner	130 (75.1)		130 (75.6)				93 (71.0)		109 (77.3)				151 (87.3)					147 (85.5)						109 (85.8)			124 (90.5)								
	Children	1 (0.6)		0 (0)				1 (0.8)		0 (0)				2 (1.2)					6 (3.5)						1 (0.8)			0 (0)								
	Other	1 (0.6)		0 (0)				2 (1.5)		7 (5.0)				2 (1.2)					1 (0.6)						4 (3.1)			6 (4.4)								

^a^MCI: mild cognitive impairment.

^b^n=130 people with MCI in the intervention group at follow-up; n=125 carers in the intervention group at follow-up.

### Cost Measures

The average per-person cost for people with MCI was €7032 in the intervention group and €7024 in the control group over the 18-month period (see Table S5 in Multimedia Appendix 2). Outpatient care accounted for the majority of health care costs in both groups, representing total costs of €5466 (77.73%) in the intervention group and €5639 (80.28%) in the control group. The average cost per informal caregiver in the intervention group was €5371, while in the control group it was €4903. In contrast to the intervention group, informal caregivers in the control group had higher outpatient and lower inpatient costs. However, no statistically significant differences were observed between the 2 groups (*P*=.63).

### Effect Measures

Over the 18-month period, people with MCI in both groups experienced a statistically significant decline (*P*<.001 in both groups) in their quality of life (composite QoL-AD score). However, there was a significant improvement in MMSE scores for people with MCI in both groups (*P*<.001 in the intervention group and *P*=.02 in the control group; see Table S6 in Multimedia Appendix 2). Informal caregivers in both groups also experienced a decline in their quality of life (EQ-5D-3L index score) over the study period; however, this decline was statistically significant only in the control group (*P*<.001). The inverse ZBI-12 score indicated an insignificant decrease (*P*=.52) in caregivers’ burden in the intervention group after 18 months. It further illustrates that dyads in the control group experienced a statistically significant decline in quality of life (EQ-5D-3L index score; *P*=.001) over the 18-month period. Intergroup differences for all effect measures were insignificant (for people with MCI: EQ-5D-3L, *P*=.72; composite QoL-AD, *P*=.73; for informal caregiver: EQ-5D-3L, *P*=.07; ZBI-12, *P*=.25; and for dyad: EQ-5D-3L, *P*=.25), except for MMSE scores, where the intervention group showed a statistically significant improvement (*P*=.03) compared with the control group.

### Cost-Effectiveness Analysis

**Figure 1:**
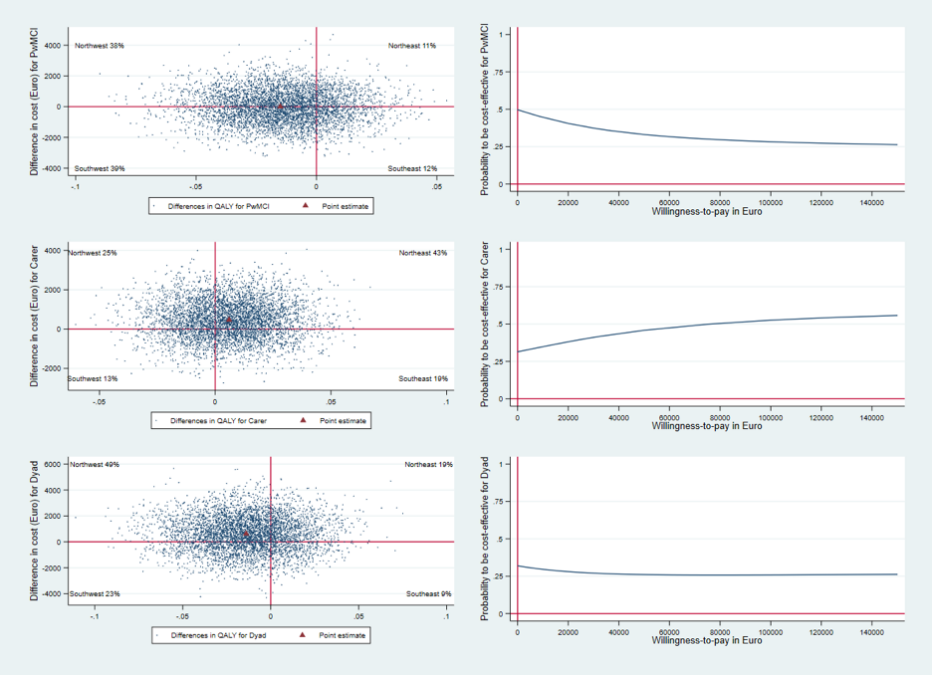
Cost-effectiveness (CE) plane from the health care provider perspective and cost-effectiveness acceptability curve (CEAC), showing the probability that Support, Monitoring, and Reminder Technology for Mild Dementia (SMART4MD) is cost-effective across different willingness-to-pay (€) thresholds per quality-adjusted life-year (QALY) gained.

The intervention was dominated by standard care for people with MCI (Table 2), as the intervention group had higher costs (€9) and lower QALYs (−0.015) compared with the control group. However, the differences were not statistically significant for either costs (*P*=.99) or QALYs (*P*=.46). Additionally, the negative NMB (–€742) suggests that the intervention was not cost-effective. Of the 5000 incremental CE pairs for QALYs, 1908 (38.16%) were in the northwest quadrant (more costly and less effective) of the CE plane (Figure 1). At a WTP of €48,876 per QALY, the CEAC indicated an approximately 30% likelihood of the intervention being cost-effective (Figure 1). The ICERs for the composite QoL-AD and MMSE scores were €56 and €15 per unit gain, respectively.

For informal caregivers, the intervention group demonstrated an improvement in QALYs (0.006) at higher costs (€468) compared with the control group. The resulting ICER of €78,000 per QALY and a negative NMB (−€175) indicate that the intervention is not cost-effective at the WTP threshold of €48,876 per QALY. On the CE plane, 2157 out of 5000 (43.14%) CE pairs were in the northeast quadrant, indicating higher costs and higher effectiveness. The CEAC indicated that the likelihood of the intervention being cost-effective for caregivers was approximately 48% at a WTP of €48,876 per QALY (Figure 1). Moreover, due to a reduction in caregiving burden in the intervention group compared with the control group, the ICER for ZBI-12 was €613 per unit reduction in caregiving burden.

Table 2 shows higher costs (€630) and lower QALYs (−0.014) for dyads in the intervention group compared with the control group. These results, along with the negative NMB (−€1314), indicate that the intervention is dominated by standard care. The probability of the intervention being cost-effective at a WTP of €48,876 was estimated to be around 25% (Figure 1).

**Table 2:** Differences in pooled mean cost and health effects with 95% CI, ICERs^a^, and NMB^b^ (€^c^)—BTH^d^.^e^

Participants			Costs									Effects									ICERs				NMB	
			Sample size^f^ (intervention/control), n		Intervention, mean (SD)		Control, mean (SD)		ΔC (Bootstrap 95% CI)		Sample size^f^ (intervention/control), n			Intervention, mean (SD)		Control, mean (SD)		ΔE (Bootstrap 95% CI)								
**People with mild cognitive impairment**																										
	Aggregated quality-adjusted life-years	128/143		7032 (9272)		7024 (7976)		9 (–2082 to 2099)		127/143			1.282 (0.16)		1.297 (0.16)		–0.015 (–0.05 to 0.02)		Dominated				–742			
	Composite quality of life in Alzheimer disease	128/143		7032 (9272)		7024 (7976)		9 (–2082 to 2099)		120/133			–1.525 (3.56)		–1.687 (3.82)		0.162 (–0.75 to 1.07)		56				N/A^g^			
	Mini-Mental State Examination	128/143		7032 (9272)		7024 (7976)		9 (–2082 to 2099)		130/141			1.046 (2.37)		0.454		0.592 (0.06 to 1.12)		15				N/A			
**Carer**																										
	Quality-adjusted life years	123/135		5371 (8509)		4903 (7014)		468 (–1432 to 2368)		122/132			1.299 (0.13)		1.292 (0.15)		0.006 (–0.03 to 0.04)		78,000				–175			
	12-item Zarit Burden Interview	123/135		5371 (8509)		4903 (7014)		468 (–1432 to 2368)		126/136			0.286 (4.94)		–0.478 (5.63)		0.764 (–0.51 to 2.04)		613				N/A			
**Dyad**																										
	Quality-adjusted life years	123/135		11,877 (12,566)		11,247 (8810)		630 (–2038 to 3299)		121/132			2.590 (0.19)		2.604 (0.19)		–0.014 (–0.06 to 0.03)		Dominated				–1314			

^a^ICER: incremental cost-effectiveness ratio.

^b^NMB: net monetary benefit.

^c^€1=US $1.16.

^d^BTH: Blekinge Institute of Technology.

^e^Incremental effect with a positive value represents improved outcomes. We reversed the 12-item Zarit Burden Interview scores to obtain this. Deceased people with mild cognitive impairment are included in quality-adjusted life years and cost calculations. The incremental costs for dyads do not add up to the individual with mild cognitive impairment and informal caregivers cost differences due to different sample sizes.

^f^The number of participants available in the intervention group is followed by the number of participants available in the control group.

^g^N/A: not applicable.

### Sensitivity and Subgroup Analyses

Table 3 presents the results of the sensitivity and subgroup analyses. While numerical changes in costs and effects were observed across different analyses, the base-case findings remained generally consistent. Some exceptions were observed. Sensitivity analyses for people with MCI older than 70 years and with MMSE ≤26 showed that the intervention was less costly and less effective. For informal caregivers, the intervention was dominant among low app users. Further, the intervention was cost-effective for informal caregivers older than 70 years, female informal caregivers, those with health care costs greater than zero, and when QALYs were imputed.

**Table 3:** Sensitivity analyses from the health care provider perspective in ICERs^a^—Blekinge Institute of Technology.^b^

	Number	Scenarios	Sample size^c^, n	Changes in cost (bootstrap 95% CI)	Changes in effect (quality-adjusted life-years; bootstrap 95% CI)	ICER (€^d^)	
	Intervention	Control					
**People with mild cognitive impairment**							
	Base case	128/127	143/143	9 (–2082 to 2099)	–0.015 (–0.05 to 0.02)	Dominated	
	1. Imputed quality-adjusted life-years change	128/173	143/172	9 (–2082 to 2099)	–0.003 (–0.04 to 0.03)	Dominated	
	2. With intervention cost	128/127	143/143	182 (–1909 to 2272)	–0.015 (–0.05 to 0.02)	Dominated	
	3. Removing zero health care cost	122/121	138/138	100 (–2013 to 2213)	–0.014 (–0.05 to 0.03)	Dominated	
	4a. Men only	74/74	89/88	–445 (–3162 to 2272)	–0.010 (–0.06 to 0.04)	44,500	
	4b. Women only	54/53	54/54	824 (–2310 to 3958)	–0.017 (–0.07 to 0.03)	Dominated	
	5a. Age ≤70 years	18/18	19/19	1484 (–2057 to 5025)	0.028 (–0.03 to 0.09)	53,000	
	5b. Age >70 years	110/109	124/124	–204 (–2473 to 2065)	–0.022 (–0.06 to 0.02)	9273	
	6a. Mini-Mental State Examination score ≤26	48/47	39/39	–1783 (–5028 to 1461)	–0.016 (–0.09 to 0.06)	111,438	
	6b. Mini-Mental State Examination score >26	80/80	104/104	853 (–1863 to 3569)	–0.008 (–0.05 to 0.03)	Dominated	
	7a. High app use >125	66/65	143/143	–159 (–2751 to 2434)	–0.002 (–0.04 to 0.04)	79,500	
	7b. Low app use ≤125	61/61	143/143	158 (–2504 to 2819)	–0.007 (–0.04 to 0.03)	Dominated	
**Informal caregiver**							
	Base case	123/122	135/132	468 (–1432 to 2368)	0.006 (–0.03 to 0.04)	78,000	
	1. Imputed quality-adjusted life-years change	123/173	135/170	468 (–1432 to 2368)	0.011 (–0.02 to 0.04)	42,545	
	2. Removing zero health care cost	110/109	116/114	300 (–1807 to 2406)	0.012 (–0.03 to 0.05)	25,000	
	3a. Men only	39/39	39/38	960 (–2969 to 4888)	–0.005 (–0.04 to 0.03)	Dominated	
	3b. Women only	84/83	96/94	254 (–1932 to 2440)	0.008 (–0.04 to 0.05)	31,750	
	4a. Age ≤70 years	55/55	57/56	663 (–1231 to 2558)	–0.024 (–0.08 to 0.03)	Dominated	
	4b. Age >70 years	68/67	78/76	435 (–2579 to 3449)	0.028 (–0.01 to 0.07)	15,536	
	5a. 12-Item Zarit Burden Interview score >31 (no burden)	117/116	125/122	631 (–1369 to 2632)	0.009 (–0.03 to 0.04)	70,111	
	5b. 12-Item Zarit Burden Interview score ≤31 (some burden)	6/6	10/10	–2378 (–5722 to 966)	–0.069 (–0.19 to 0.05)	34,464	
	6a. High app use >125	64/63	135/132	1528 (–1215 to 4271)	–0.003 (–0.04 to 0.04)	Dominated	
	6b. Low app use ≤125	59/59	135/132	–682 (–2603 to 1240)	0.016 (–0.03 to 0.06)	Dominant	
**Dyads (people with mild cognitive impairment plus an informal caregiver)**							
	Base case	123/121	135/132	630 (–2038 to 3299)	–0.014 (–0.06 to 0.03)	Dominated	
	1. Imputed quality-adjusted life-years change	123/173	135/170	630 (–2038 to 3299)	0.008 (–0.04 to 0.05)	78,750	
	2. With intervention cost	123/121	135/132	804 (–1865 to 3472)	–0.014 (–0.06 to 0.03)	Dominated	
	3. Removing zero health care cost	123/121	134/131	547 (–2149 to 3243)	–0.013 (–0.06 to 0.03)	Dominated	

^a^ICER: incremental cost-effectiveness ratio.

^b^Incremental effect with positive value represents improved outcomes. We reversed 12-Item Zarit Burden Interview scores to obtain this.

^c^Number of participants available for cost estimation first, followed by the number of participants available for health effects.

^d^€1=US $1.16.

### Scenario Analyses

Results for the scenario analysis of people with MCI who participated in the CST site are presented in Table 4. The intervention was more costly and more effective in terms of QALYs, with an ICER of €3653/QALY and an NMB of €1897, indicating that the intervention is cost-effective at the threshold of €30,000/QALY. The results for the composite QoL-AD score showed that the intervention was more costly and more effective, with an ICER of €17,533 per unit gain. However, a negative effect of the intervention in terms of MMSE indicates that the intervention was dominated by standard care. All differences in costs and outcomes between the intervention and control groups were statistically insignificant (difference in cost, *P*=.66; QALY Spain, *P*=.29; QoL-AD, .98; and MMSE, .55). Further, intergroup differences in demographic characteristics and health outcomes for the Spanish site (CST) are presented in Table S7 in Multimedia Appendix 2. The MMSE score was statistically significant at baseline (*P*=.04), indicating that people with MCI in the intervention group had lower cognitive abilities than those in the control group.

The results of the scenario analyses for people with MCI from both the BTH and CST sites indicated that the intervention was less costly and more effective compared with the control group, as measured by QALYs, QoL-AD, and MMSE scores.

**Table 4:** Scenario analyses of differences in pooled mean cost and health effects for people with mild cognitive impairment with 95% CI, ICERs^a^, and NMB^b^ (€^c^).^d^

Scenario analysis			Costs									Health effects												
			Sample size^e^ (intervention/control), n		Intervention, mean (SD)		Control, mean (SD)		ΔC (bootstrap 95% CI)		Sample size^e^ (intervention/control), n			Intervention, mean (SD)		Control, mean (SD)		ΔE (bootstrap 95% CI)		ICERs			NMB	
**Consorci Sanitaria de Terrassa**																								
	Mean quality-adjusted life-years	70/69		1192 (4023)		929 (3051)		263 (–918 to 1444)		69/67			1.104 (0.41)		1.033 (0.41)		0.072 (–0.06 to 0.21)		3653			1897		
	Quality of life in Alzheimer disease	70/69		1192 (4023)		929 (3051)		263 (–918 to 1444)		68/75			0.211 (3.84)		0.196 (3.34)		0.015 (–1.17 to 1.20)		17533			N/A^f^		
	Mini-Mental State Examination	70/69		1192 (4023)		929 (3051)		263 (–918 to 1444)		73/80			–1.274 (4.05)		–0.913 (3.44)		–0.361 (–1.54 to 0.82)		Dominated			N/A		
**Consorci Sanitaria de Terrassa plus Blekinge Institute of Technology**																								
	Mean quality-adjusted life-years	198/212		4968 (8302)		5040 (7349)		–72 (–1585 to 1440)		196/210			1.122 (0.26)		1.121 (0.26)		0.00021 (–0.05 to 0.05)		Dominant			2232		
	Quality of life in Alzheimer disease	198/212		4968 (8302)		5040 (7349)		–72 (–1585 to 1440)		188/208			–0.897 (3.75)		–1.008 (3.76)		0.111 (–0.63 to 0.85)		Dominant			N/A		
	Mini-Mental State Examination	198/212		4968 (8302)		5040 (7349)		–72 (–1585 to 1440)		203/221			0.21 (3.27)		–0.04 (2.79)		0.25 (–0.33 to 0.83)		Dominant			N/A		

^a^ICER: incremental cost-effectiveness ratio.

^b^NMB: net monetary benefit.

^c^€1=US $1.16.

^d^No significant differences in incremental costs and effects were found (independent *t* test).

^e^Number of participants available for cost estimation first, followed by number of participants available for health effects.

^f^N/A: not applicable.

## Discussion

### Principal Findings

We performed an economic evaluation of the SMART4MD trial from a health care provider’s perspective over the trial duration. Our findings indicate that, except for MMSE scores, there were no significant differences in effects and costs between the intervention and control groups. The estimated cost-effectiveness ratios show that the intervention is dominated by standard care for both people with MCI and dyads, and it was not found to be cost-effective for informal caregivers when QALYs were the outcome. However, for the Spanish site, the intervention was cost-effective, with an ICER of €3337/QALY.

In the base-case analysis, the intervention was not cost-effective in Sweden at the country-specific WTP threshold, whereas it was cost-effective in Spain. This divergence is likely driven primarily by structural and contextual differences between settings. First, country-specific cost inputs played an important role: higher unit costs of health care utilization and intervention delivery in Sweden increased incremental costs relative to usual care, while lower unit costs in Spain resulted in smaller incremental costs. Second, baseline risk and disease severity differed between countries, with the Spanish population exhibiting a less favorable risk profile; consequently, the same relative treatment effect translated into larger absolute health gains and more favorable ICERs in Spain. Third, variation in usual care meant that more intensive standard care in Sweden reduced the marginal benefit of the intervention, whereas in Spain, a less intensive usual care comparator allowed greater incremental benefit. Taken together, these factors suggest that the observed cross-country differences in cost-effectiveness are driven more by contextual variation in costs, baseline risk, and usual care than by differences in the intrinsic effectiveness of the intervention itself, underscoring the need for caution when extrapolating our findings to health systems with substantially different cost structures and treatment pathways.

The findings of this study for people with MCI concur with our previously published short-term (6-month) economic evaluation of the SMART4MD trial [12]. Both studies indicate that the intervention is dominated by standard care for people with MCI and dyads in terms of QALYs and is not cost-effective for informal caregivers at a WTP threshold of €48,876 per QALY [12]. While the ICER for people with MCI, when aggregating data from the Swedish and Spanish sites, indicates a dominant point estimate, the differences in costs and QALYs were not statistically significant.

It is well established in the literature that using the EQ-5D-3L instrument might not be the most suitable approach for assessing the quality of life of people with dementia. This is because the EQ-5D-3L does not encompass attributes that adequately capture cognitive function [44], relationships with caregivers, and social support, which are recognized as important factors in measuring quality of life for people with dementia [45]. However, we also included other health outcome measures, such as MMSE scores, QoL-AD, and caregiving burden (ZBI-12 score), to cover these aspects to some extent and complement the results of the primary outcome measure.

For people with MCI, the ICERs for the remaining 2 outcome measures were €55 per unit gain in composite QoL-AD and €15 per unit gain in MMSE score, respectively. It is uncertain whether these values can be deemed cost-effective due to the absence of established cost-effectiveness thresholds for QoL-AD and MMSE. However, low ICER values may indicate that the societal value associated with a 1-unit change in these outcome measures is likely to be higher.

Similar to previous effectiveness and cost-effectiveness studies on the SMART4MD trial [12,46], this study also indicates an improvement in the quality of life of informal caregivers in the intervention group compared with the control group. This may suggest that informal caregivers gained the most from the intervention, even though it was intended to be used primarily by people with MCI themselves. It is possible that the app served as a reminder for informal caregivers regarding medication schedules and doctors’ appointments for people with MCI. Furthermore, it is important to acknowledge that the involvement of multiple informal caregivers for an individual with dementia may enhance the benefits of SMART4MD, potentially leading to an underestimation of its impact.

The available research on the cost-effectiveness of mHealth interventions aimed at older adults with chronic conditions in a home-care setting is inconclusive [47]. For example, some studies have shown that mHealth interventions for patients with chronic obstructive pulmonary disease in the United Kingdom [48] and in Denmark [49] were not cost-effective. However, mHealth interventions were deemed cost-effective for older adults diagnosed with Parkinson disease [50] and for cardiac rehabilitation [42]. Therefore, comprehensive evidence regarding the cost-effectiveness of mHealth for older adults living with chronic conditions in general, and with MCI or dementia in particular, appears to be lacking. The generalizability of a specific mHealth intervention is also limited due to several factors, such as the learning curve of health care professionals and participants [48,49], the level of engagement of health care professionals [49], and participants’ adherence rates [50].

In our previous study [12], we reasoned that the cost-effectiveness of the SMART4MD intervention might be underestimated due to the short (6-month) follow-up period. This study, conducted over a longer follow-up period of 18 months, still did not find significant differences between the intervention and control groups, especially with respect to QoL outcome measures. One reason could be that the 18-month period may still not be long enough to capture the full health effects. A model-based economic evaluation could be used to extrapolate the health effects of such an intervention over a lifetime horizon, which was beyond the scope of this study. By contrast, short follow-up periods are common in dementia research due to its rapid progression [51]. Thus, it is possible that the SMART4MD app is not a cost-effective intervention, as suggested by the results of our study. However, the results also show a statistically significant improvement in MMSE scores for people with MCI in the intervention group compared with the control group. The insignificant results for QoL outcome measures might be due to an underpowered trial with respect to cost-effectiveness analysis. The sample size of this trial was based on clinical effectiveness rather than cost-effectiveness. However, it is important to note that no significant differences were observed for QoL-AD, which was the primary outcome measure of the trial and was used to calculate the sample size [11].

A high dropout rate is a common issue when mHealth interventions are introduced [52], and the SMART4MD trial is no exception. At the 18-month follow-up, 42 of the 173 (24.2%) participants in the intervention group and 31 of the 172 (18.0%) in the control group had left the trial. As previously discussed [12,46], a potential explanation for leaving the trial could be the perceived burden of participation. However, a previously published qualitative study, using individual semistructured interviews, was conducted with 16 people with MCI who participated in the SMART4MD trial. The findings showed that these individuals did not perceive the app’s reminder feature as beneficial [53], potentially affecting their level of engagement with the app. This is also evident from the data, which show that only 195 of 312 (62.5%) individuals with MCI accessed the app between 6 and 12 months, compared with 299 out of 312 (95.8%) during the initial 6 months. Given that the difference in dropout rates between the groups was not statistically significant (*P*=.15), we assume that it does not have a meaningful impact on the cost-effectiveness results of this study. However, we conducted our primary analysis on an intention-to-treat basis, which preserves the benefits of randomization and reflects the effectiveness of offering the intervention under real-world patterns of uptake and adherence, including the observed decline in app use over time. In this context, a per-protocol analysis restricted to highly adherent users would likely yield more favorable ICERs but would be less representative of routine practice and more vulnerable to selection bias. We therefore did not perform such an analysis and interpret our results as estimates of cost-effectiveness under realistic, rather than optimal, adherence.

Missing cost data due to attrition were handled within the same multiple imputation framework used for missing outcomes, assuming data were missing at random, conditional on treatment group, baseline covariates, and observed intermediate costs and effects. All relevant cost components and utility measures at each time point were included in the imputation model, and imputed individual-level costs were then used to construct total costs over the trial horizon and estimate ICERs, with results pooled across imputations using rules proposed by Rubin [35]. While this approach is recommended over complete-case analysis, it still relies on untestable assumptions about the missingness mechanism; we therefore interpret the cost-effectiveness results with appropriate caution.

### Study Strengths

The study has several strengths. Given the limited evidence available on the cost-effectiveness of mHealth interventions designed for people with MCI and their informal caregivers [12], this study extends the knowledge base for this type of intervention. Moreover, this economic evaluation is based on a pragmatic RCT [54], which improves the generalizability of the findings to older adults who visit primary care facilities seeking assistance with memory-related concerns. The absence of data on medication is one limitation of this study, as it is an important component of the health care provider’s perspective. Furthermore, it is recommended in the literature to collect health care utilization data before randomization for a specific period, such as 1, 3, or 12 months. This prerandomization cost data could serve as a predictor of postrandomization costs and potentially capture variations in health care use before and after the trial [55]. Unfortunately, in our study, we did not have access to prerandomization cost data. Also, due to the absence of cost data from Spanish sites, the findings of this study relied exclusively on Swedish data; consequently, the results may not represent all participating sites. Finally, we were unable to analyze from a societal perspective due to the absence of crucial cost categories. These include the costs of social support, informal care, and participants’ out-of-pocket expenses. Considering the positive outcomes for caregivers in this study, and that informal care constitutes around 50% of the total cost of dementia [56], an analysis from a societal perspective would have been informative.

Due to a lack of data regarding the app’s actual utilization, our analysis is restricted to examining its presence, which offers people with MCI the opportunity to use it. Consequently, we also lack data on any behavioral changes associated with app usage. Therefore, the focus of this study was on enhancement in health-related quality of life, as such an improvement in QoL would suggest a behavioral change. Given the results, further investigations are necessary to determine whether the intervention failed to induce behavioral changes in participants or whether any behavioral changes observed had no impact on the primary outcome measure of health-related quality of life.

Neither the researchers nor the participants were blinded to the intervention and control groups, which could have introduced bias. However, blinding may be less critical in pragmatic trials that aim to closely replicate real-world practice [57]. Future studies could address this issue by providing an app with limited features to the control group. Another limitation was the lack of detailed characterization of informal caregivers. Key details, such as the primary caregiver’s relationship with people with MCI, caregiving hours per day or week, and employment status, were not recorded. Additionally, the impact of the SMART4MD app was not analyzed based on caregiver type (spouse, adult child, or other), which could have offered valuable insights into which group benefited most from the intervention.

### Conclusions

In conclusion, total costs and QALYs over the 18-month trial period were not significantly different between the intervention and control groups, indicating that the SMART4MD intervention was neither better nor worse than standard care. The results suggest that this type of tablet-based app for people with MCI and dyads is not cost-effective.

## Appendix

Multimedia Appendix 1 Consolidated Health Economic Evaluation Reporting Standards (CHEERS) checklist.

Multimedia Appendix 2 Additional analysis.

## Abbreviations

BTH: Blekinge Institute of Technology
CEAC: cost-effectiveness acceptability curve
CHEERS: Consolidated Health Economic Evaluation Reporting Standards
CST: Consorci Sanitaria de Terrassa
ICER: incremental cost-effectiveness ratio
MCI: mild cognitive impairment
mHealth: mobile health
MMSE: Mini-Mental State Examination
NMB: net monetary benefit
QALY: quality-adjusted life-year
QoL-AD: Quality of Life in Alzheimer’s Disease Scale
RCT: randomized controlled trial
SEK: Swedish kronor
SMART4MD: Support, Monitoring, and Reminder Technology for Mild Dementia
WTP: willingness to pay
ZBI-12: 12-item Zarit Burden Interview
