# Supplementary material for: Support, Monitoring, and Reminder Technology for Mild Dementia (SMART4MD) for People With Mild Cognitive Impairment and Their Informal Caregivers: Cost-Effectiveness Analysis
Source: JMIR Hum Factors. 2026 May 22;13:e77808. doi: 10.2196/77808 (PMC13241793; doi:10.2196/77808)
Supplement: Multimedia Appendix 1 [file humanfactors_v13i1e77808_app1.docx]

**CHEERS 2022 Checklist**

| Item no. | Section | Item (short) | Guidance (from CHEERS 2022) | Reported in this manuscript |
| --- | --- | --- | --- | --- |
| 1 | Title | Title | Identify the study as an economic evaluation and specify the interventions being compared. | Title page – identifies “cost‑effectiveness analysis” of SMART4MD app plus standard care versus standard care alone in Sweden and Spain. |
| 2 | Abstract | Abstract | Provide a structured summary that highlights context, key methods, results and alternative analyses. | Abstract – structured, gives background, objectives, perspective, time horizon, main cost and QALY results, ICERs and scenario analyses. |
| 3 | Introduction | Background and objectives | Give the context for the study, the study question and its practical relevance. | Introduction – burden of MCI/dementia in Sweden and Spain, rationale for SMART4MD, previous 6‑month evaluation, objective to assess 18‑month cost‑effectiveness from healthcare provider perspective. |
| 4 | Methods | Health economic analysis plan | Indicate whether a health economic analysis plan was developed and where available. | Methods – Analysis of cost‑effectiveness / Statistical analysis – trial‑based economic evaluation described; no separate public HEAP referenced, analysis plan summarised in text. |
| 5 | Methods | Study population | Describe characteristics of the study population. | Methods – The SMART4MD trial; Results – Table 1 – PwMCI ≥55 years with MMSE 20–28 and informal caregiver; inclusion/exclusion criteria and baseline demographics for PwMCI and caregivers. |
| 6 | Methods | Setting and location | Provide relevant contextual information that may influence findings. | Methods – The SMART4MD trial; Resource use measurement and estimating costs – Swedish site (BTH, Blekinge) and Spanish site (CST), primary and specialist care settings, registry sources. |
| 7 | Methods | Comparators | Describe the interventions or strategies being compared and why chosen. | Methods – The SMART4MD trial – intervention: SMART4MD tablet app plus standard care; comparator: standard care alone; standard care described for Sweden and Spain. |
| 8 | Methods | Perspective | State the perspective(s) adopted and why chosen. | Methods – Resource use measurement and estimating costs – healthcare provider (regional council) perspective; municipal/social care and informal care costs excluded, noted as limitation. |
| 9 | Methods | Time horizon | State the time horizon for the study and why appropriate. | Methods – The SMART4MD trial; Measurement of effectiveness – 18‑month follow‑up chosen to capture medium‑term costs and effects of the intervention. |
| 10 | Methods | Discount rate | Report the discount rate(s) and reason chosen. | Methods – Analysis of cost‑effectiveness – 3% annual discount applied to costs and QALYs in final 6 months, following Swedish and Spanish economic evaluation guidelines. |
| 11 | Methods | Selection of outcomes | Describe what outcomes were used as measures of benefit and harm. | Methods – Measurement of effectiveness – primary: QALYs from EQ‑5D‑3L for PwMCI, caregivers, and dyads; secondary: QoL‑AD, MMSE, Zarit Burden Interview (ZBI‑12, inverted). |
| 12 | Methods | Measurement of outcomes | Describe how outcomes were measured. | Methods – EQ‑5D‑3L index score; QALYs; QoL‑AD; MMSE; ZBI – describes instruments, scoring, and measurement schedule at baseline, 6, 12, and 18 months. |
| 13 | Methods | Valuation of outcomes | Describe population and methods used to measure and value outcomes. | Methods – EQ‑5D‑3L index score and QALYs – EQ‑5D‑3L utilities obtained using Swedish experience‑based tariff in base case; Spanish tariff and EVAS tariff applied in scenario analyses; AUC method for QALYs. |
| 14 | Methods | Measurement and valuation of resources and costs | Describe how costs were valued. | Methods – Resource use measurement and estimating costs – inpatient/outpatient utilisation from Swedish registers and Spanish hospital data; DRG‑based episode costs; unit costs from regional price lists; handling of missing costs; intervention costs (tablet, data, nurse time) specified and used in sensitivity analysis only. |
| 15 | Methods | Currency, price date, and conversion | Report dates of resource quantities and unit costs, currency and year of conversion. | Methods – Resource use measurement and estimating costs; Scenario analyses – Swedish costs in 2020 Euros using specified SEK/€ exchange rate; Spanish costs in 2019 Euros; price years clearly stated. |
| 16 | Methods | Rationale and description of model | If modelling is used, describe in detail and why; report availability. | Methods – Analysis of cost‑effectiveness – trial‑based economic evaluation without separate decision‑analytic model; scenario analyses (Sweden vs Spain, combined sites) described; no external model to access. |
| 17 | Methods | Analytics and assumptions | Describe methods for analysing/transforming data, extrapolation, and model validation. | Methods – Analysis of cost‑effectiveness; Statistical analysis – AUC QALYs using actual time between visits; ICER and NMB; multiple imputation for missing EQ‑5D‑3L and costs; 5,000 bootstrap resamples; CE‑planes and CEACs; no extrapolation beyond 18 months. |
| 18 | Methods | Characterizing heterogeneity | Describe methods used to estimate how results vary for sub‑groups. | Methods – Sensitivity, and subgroup analyses – pre‑specified subgroup analyses by sex, age (≤70 vs >70), baseline MMSE (≤26 vs >26), baseline Zarit burden, and app‑use frequency; separate cost‑effectiveness results for each subgroup. |
| 19 | Methods | Characterizing distributional effects | Describe how impacts are distributed or adjustments for priority populations. | Methods – Subgroup analyses and Discussion – differences in results between PwMCI, caregivers, and dyads and across subgroups discussed; no explicit equity weighting or formal distributional analysis. |
| 20 | Methods | Characterizing uncertainty | Describe methods to characterise sources of uncertainty. | Methods – Analysis of cost‑effectiveness – non‑parametric bootstrap (5,000 resamples) for ICERs; CE‑planes and CEACs; sensitivity analyses on intervention costs, zero‑cost exclusions, tariffs, and scenarios. |
| 21 | Methods | Approach to engagement with patients and others affected | Describe approaches to engage patients, public, or stakeholders. | Methods – The SMART4MD trial; Introduction – SMART4MD app developed in consultation with PwMCI, informal caregivers, and healthcare professionals; economic analysis itself not co‑designed but built on this user‑informed intervention. |
| 22 | Results | Study parameters | Report all analytic inputs including uncertainty/distributional assumptions. | Results – Cost measures; Effect measures; Table 1; Table 2 – mean costs, QALYs, QoL‑AD, MMSE, ZBI, and their differences with 95% CIs; WTP thresholds described in Methods. |
| 23 | Results | Summary of main results | Report mean values for main cost and outcome categories, summarised in overall measure. | Results – Cost‑Effectiveness Analysis – incremental costs, QALYs, ICERs, and NMB for PwMCI, caregivers, and dyads; scenario results for Spanish site and combined sites. |
| 24 | Results | Effect of uncertainty | Describe how uncertainty about judgments, inputs, or projections affects findings. | Results – Cost‑Effectiveness Analysis; Sensitivity, and subgroup analyses – CE‑planes, CEACs, and sensitivity/subgroup analyses show probability of cost‑effectiveness under different assumptions and WTP thresholds; discount rate and horizon held at guideline values. |
| 25 | Results | Effect of engagement with patients and others | Report any difference patient/stakeholder involvement made to approach or findings. | Discussion – user involvement in app design acknowledged; no specific changes to economic methods attributed to patient involvement, and this is not quantified in results. |
| 26 | Discussion | Study findings, limitations, generalizability, and current knowledge | Report key findings, limitations, ethical or equity considerations, and impact. | Discussion and Conclusions – non‑cost‑effective results for Swedish PwMCI, caregivers, and dyads; more favourable Spanish scenario; limitations (EQ‑5D‑3L suitability, missing data, lack of societal perspective, short horizon, limited power, adherence) and implications for digital dementia care and future research. |
| 27 | Other | Source of funding | Describe funding and any funder role. | Funding / Acknowledgements – EU Horizon 2020 SMART4MD and related funding sources listed; funders had no role in design, data collection, analysis, or manuscript decision. |
| 28 | Other | Conflicts of interest | Report authors’ conflicts of interest. | Conflicts of interest / Competing interests – authors state they have no competing interests. |
