# Supplementary material for: Support, Monitoring, and Reminder Technology for Mild Dementia (SMART4MD) for People With Mild Cognitive Impairment and Their Informal Caregivers: Cost-Effectiveness Analysis
Source: JMIR Hum Factors. 2026 May 22;13:e77808. doi: 10.2196/77808 (PMC13241793; doi:10.2196/77808)
Supplement: Multimedia Appendix 2 [file humanfactors_v13i1e77808_app2.docx]

Supplementary Material


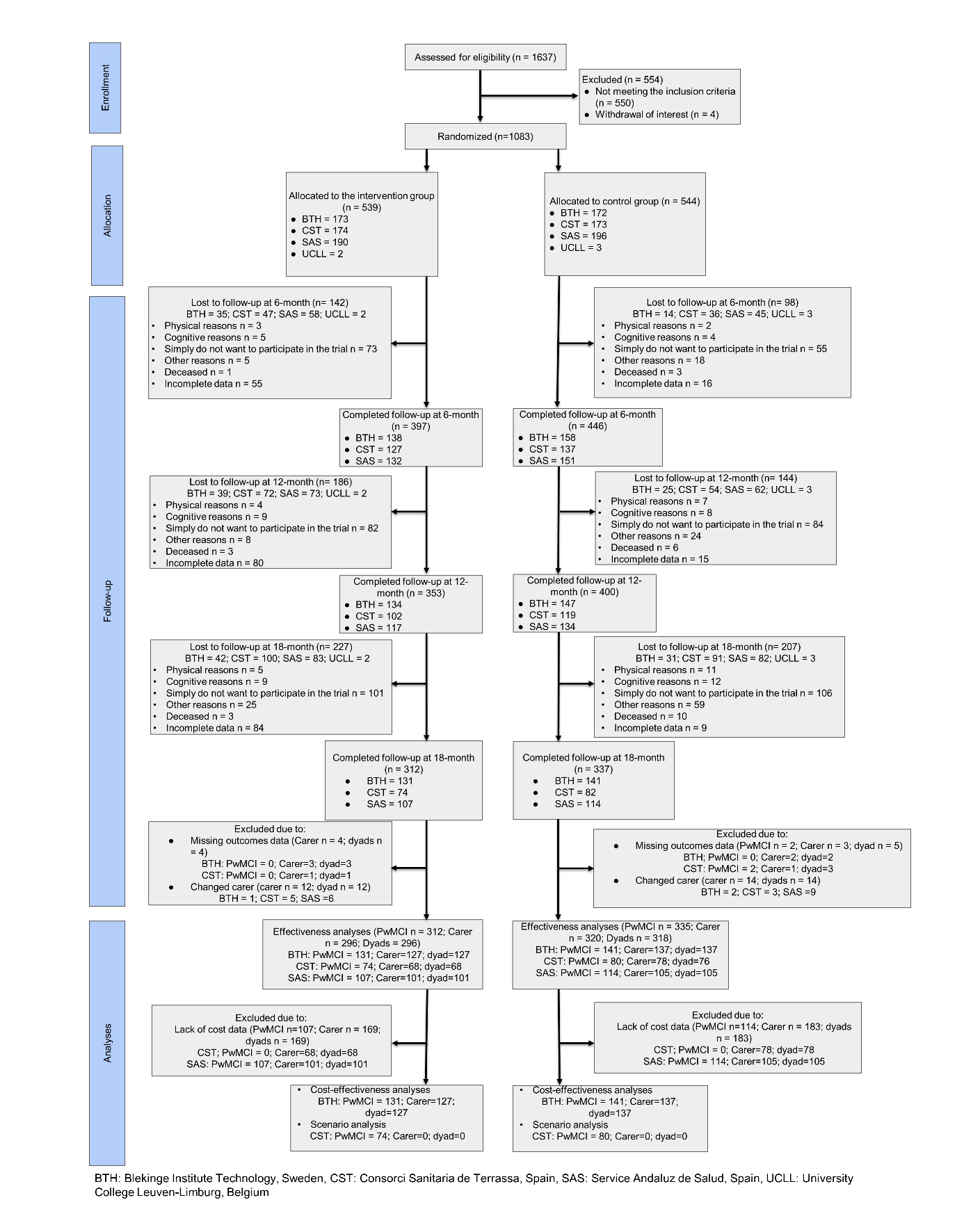


Supplementary Figure 1: Flow-chart of the dyad’s participation

| **Supplementary Table 1**  *Unit Costs in SEK/visit to specialized out-patient clinics and primary care* | | | | | | | |
| --- | --- | --- | --- | --- | --- | --- | --- |
|  |  |  | **2017** | **2018** | **2019** | **2020** | **2021** |
| **1** | Infection | Doctor | 2 248 | 2 631 | 2 704 | 3 051 | 3 193 |
|  |  | Nurse | 1 321 | 1 362 | 1 524 | 1 459 | 2 326 |
|  |  | Other profession | 1 310 | 1 684 | 1 409 | 1 447 | 1 774 |
| **2** | Surgical Clinic | Doctor | 4 479 | 5 989 | 5 534 | 7 397 | 8 166 |
|  |  | Nurse | 2 822 | 4 295 | 4 732 | 4 467 | 5 007 |
|  |  | Other profession | 3 040 | 3 672 | 735 | 819 | 1 610 |
| **3** | Medicine / Rehab | Doctor | 4 854 | 5 181 | 4 925 | 7 264 | 7 854 |
|  |  | Nurse | 3 655 | 3 916 | 4 539 | 4 797 | 5 212 |
|  |  | Other profession | 2 562 | 2 817 | 3 580 | 4 852 | 5 345 |
| **4** | Orthopedics clinic | Doctor | 3 487 | 3 677 | 3 651 | 4 467 | 4 748 |
|  |  | Nurse | 1 980 | 2 109 | 2 305 | 2 804 | 3 022 |
|  |  | Other profession |  |  | 1 355 | 1 611 | 1 530 |
| **5** | Primary care | Doctor | 1200 | 1200 | 1 647 | 2764 | 2023 |
|  |  | Nurse | 700 | 700 | 1 182 | 1792 | 1299 |
|  |  | Other profession | 700 | 700 | 732 | 1405 | 1547 |
| **6** | Thorax Center | Doctor | 5 644 | 6 253 | 5 908 | 5 414 | 7 380 |
|  |  | Nurse | 2 722 | 2 677 | 3 604 | 4 122 | 3 128 |
|  |  | Other profession | 3 958 | 3 642 | 4 184 | 3 192 | 3 339 |
| **7** | Adult psychiatry | Doctor | 5 023 | 5 025 | 5 341 | 5 964 | 6 064 |
|  |  | Nurse | 1 754 | 1 954 | 1 781 | 1 972 | 2 242 |
|  |  | Other profession | 1 959 | 2 370 | 2 482 | 2 754 | 2 899 |
| **8** | Eye clinic | Doctor | 2 868 | 2 762 | 3 350 | 3 621 | 3 679 |
|  |  | Nurse | 1 452 | 1 594 | 1 444 | 2 021 | 1 942 |
|  |  | Other profession | 1 916 | 2 123 | 3 626 | 3 533 | 2 366 |
| **9** | Ear- nose-throat | Doctor | 3 481 | 3481 | 3 481 | 3 481 | 4099 |
|  |  | Nurse | 2 600 | 2 600 | 2 600 | 2 600 | 2 600 |
|  |  | Other profession | 2 906 | 2 906 | 2 906 | 2 906 | 2057 |
| **10** | Gynecology clinic | Doctor | 3 995 | 4 773 | 5 560 | 6 589 | 6 470 |
|  |  | Nurse |  |  | 4 082 | 5 843 | 5 858 |
|  |  | Other profession | 1 523 | 1 492 | 1 436 | 1 220 | 2 545 |
| **11** | Anesthesia | Doctor |  |  |  |  |  |
|  |  | Nurse | 3 679 | 2 974 | 4 861 | 2 528 | 6493 |
|  |  | Other profession |  |  |  |  |  |
| **12** | Palliative medicine | Doctor |  |  | 15 478 | 16 179 | 18 182 |
|  |  | Nurse |  |  | 6 861 | 5 095 | 6 047 |
|  |  | Other profession |  |  | 6 372 | 4 039 | 3 194 |

| Supplementary Table 2  *Effect measures of PwMCI and informal caregivers at each time point* | | | | | | | | | | | | | | | | |
| --- | --- | --- | --- | --- | --- | --- | --- | --- | --- | --- | --- | --- | --- | --- | --- | --- |
|  | Baseline | | | | 6-month follow up | | | | 12-month follow up | | | | 18-month follow up | | | |
|  | Sample size (I/C) | Intervention | Control | Difference (*P* value) | Sample size (I/C) | Intervention | Control | Difference (*P* value) | Sample size (I/C) | Intervention) | Control | Difference (*P* value) | Sample size (I/C) | Intervention | Control | Difference (*P* value) |
| PwMCI | | | | | | | | | | | | | | | | |
| EQ-5D-3L index score^a^ | 173/172 | 0.894 (0.08) | 0.886 (0.09) | 0.008 (.40) | 138/158 | 0.869 (0.10) | 0.876 (0.10) | -0.007 (.55) | 134/147 | 0.873 (0.10) | 0.873 (0.10) | 0.000 (.96) | 130/141 | 0.887 (0.09) | 0.884 (0.10) | 0.003 (.81) |
| QALY^a, b^ |  |  |  |  | 139/158 | 0.427 (0.07) | 0.433 (0.05) | -0.004 (.58) | 130/146 | 0.431  (0.08) | 0.449 (0.07) | -0.018 (.047) | 130/145 | 0.426 (0.08) | 0.411 (0.09) | 0.015 (.17) |
| Composite QoL-AD^a^ | 173/171 | 40.171 (5.07) | 40.302 (5.02) | -0.131 (.81) | 133/154 | 39.424 (4.39) | 39.121 (4.99) | 0.302 (.59) | 121/129 | 39.047 (4.69) | 39.147 (4.51) | -0.100 (.86) | 120/134 | 38.903 (4.83) | 38.821 (4.75) | 0.082 (.89) |
| MMSE score^a^ | 173/172 | 26.526 (1.79) | 26.779 (1.64) | -0.253 (.17) | 138/158 | 27.688 (2.18) | 27.418 (2.46) | 0.271 (.32) | 134/147 | 27.694 (2.55) | 27.571 (2.66) | 0.123 (.69) | 130/141 | 27.700 (2.68) | 27.454 (2.80) | 0.246 (.46) |
| Informal Caregiver | | | | | | | | | | | | | | | | |
| EQ-5D-3L index score^a^ | 173/171 | 0.898 (0.08) | 0.890 (0.09) | 0.009 (.36) | 134/154 | 0.884 (0.11) | 0.869 (0.12) | 0.015 (.27) | 129/141 | 0.868 (0.11) | 0.869 (0.11) | 0.001 (.96) | 127/137 | 0.890 (0.09) | 0.860 (0.12) | 0.030 (.020) |
| QALY^a^ |  |  |  |  | 134/153 | 0.434 (0.07) | 0.430 (0.06) | 0.004 (.58) | 124/139 | 0.435 (0.07) | 0.447 (0.07) | -0.013 (.13) | 125/135 | 0.429 (0.08) | 0.415 (0.07) | 0.014 (.13) |
| ZBI^a^ | 173/171 | 43.526 (6.12) | 43.135 (6.61) | 0.392 (.57) | 134/154 | 43.373 (6.46) | 43.097 (6.54) | 0.276 (.72) | 129/142 | 44.318 (5.03) | 43.859 (6.17) | 0.459 (.51) | 126/137 | 44.040 (5.90) | 43.241 (5.70) | 0.799 (.27) |
| Dyad | | | | | | | | | | | | | | | | |
| EQ-5D-3L index score^a^ | 173/171 | 1.792 (0.12) | 1.775 (0.13) | 0.017 (.22) | 134/154 | 1.754 (0.15) | 1.744 (0.15) | 0.010 (.59) | 129/141 | 1.740 (0.17) | 1.742 (0.14) | -0.002 (.92) | 126/137 | 1.780 (0.13) | 1.741 (0.15) | 0.038 (.029) |
| QALY^a^ |  |  |  |  | 134/153 | 0.864 (0.11) | 0.862 (0.09) | 0.002 (.83) | 124/139 | 0.868 (0.13) | 0.898 (0.10) | -0.030 (.032) | 124/135 | 0.860 (0.14) | 0.838 (0.11) | 0.022 (.17) |
| Abbreviations*:* MMSE mini-mental state examination; PwMCI: person with mild cognitive impairment; QoL-AD: quality of life in Alzheimer disease; ZBI: Zarit Caregiver Burden Inventory.  *Note:* a: Mean (standard deviation); b: deceased included in the QALY calculation. | | | | | | | | | | | | | | | | |

| **Supplementary Table 3**  *Differences in baseline characteristics of dropout and non-dropout PwMCI at 18-month follow-up--BTH* | | | | | | |
| --- | --- | --- | --- | --- | --- | --- |
| Characteristics | Intervention Group | | | Control Group | | |
|  | Dropout (n=42) | Non-dropout (n=131) | *P* value | Dropout (n=31) | Non-dropout (n=141) | *P* value |
| Age^a^ | 77 (4.98) | 76 (5.07) | .16 | 77 (5.64) | 76 (5.08) | .35 |
| Gender, n (%) | | | | | | |
| Male | 21 (50) | 76 (58) | .36 | 16 (52) | 87 (62) | .30 |
| Female | 21 (50) | 55 (42) |  | 15 (48) | 54 (38) |  |
| Education^b^, n (%) | | | | | | |
| Elementary education | 10 (24) | 47 (36) | .15 | 12 (39) | 52 (37) | .08 |
| Secondary education | 13 (31) | 44 (34) |  | 11 (35) | 27 (19) |  |
| Higher education | 19 (45) | 39 (30) |  | 8 (26) | 62 (44) |  |
| Marital Status, n (%) | | | | | | |
| Unmarried | 10 (24) | 36 (27) | .64 | 11 (35) | 31 (22) | .11 |
| Married/Partner | 32 (76) | 95 (73) |  | 20 (65) | 110 (78) |  |
| Living arrangements, n (%) | | | | | | |
| Living alone | 8 (19) | 33 (25) | .26 | 12 (39) | 30 (21) | .04 |
| Spouse/Common law partner | 33 (79) | 97 (74) |  | 19 (61) | 111 (79) |  |
| Children | 0 (0) | 1 (1) |  | 0 (0) | 0 (0) |  |
| Other | 1 (2) | 0 (0) |  | 0 (0) | 0 (0) |  |
| EQ-5D-3L index scores ^a^ | 0.87 (0.09) * | 0.90 (0.08) | .03 | 0.85 (0.11) | 0.89 (0.09) | .0097 |
| QoL-AD ^a^ | 40.76 (6.48) | 40.49 (4.97) | .77 | 39.77 (6.44) | 41.14 (5.15) | .21 |
| MMSE score^a^ | 26.14 (2.24) | 26.65(1.61) | .11 | 25.77 (2.08) | 27.00 (1.45) | <.001 |
| Abbreviations: MMSE mini-mental state examination; PwMCI: person with mild cognitive impairment; QoL-AD: quality of life in Alzheimer disease; n number; % percentage  Note*:* Independent sample t-test is used to assess the statistical differences between dropouts and non-dropouts (inter-group [between groups] analysis).  a: Mean (standard deviation); b: n=130 for non-dropout in the intervention group. | | | | | | |

| **Supplementary Table 4**  *Differences in baseline characteristics of dropout and non-dropout informal caregiver at 18-month follow-up--BTH* | | | | | | |
| --- | --- | --- | --- | --- | --- | --- |
| Characteristics | Intervention Group | | | Control Group | | |
|  | Dropouts (n=42) | Non-dropouts (n=131) | *P* value | Dropouts (n=31) | Non-dropouts (n=141) | *P* value |
| Age^a^ | 72 (10.91) | 69 (10.28) | .20 | 68 (12.43) | 70 (11.12) | .40 |
| Gender, n (%) | | | | | | |
| Male | 17 (40) | 40 (31) | .23 | 12 (39) | 41 (29) | .29 |
| Female | 25 (60) | 91 (69) |  | 19 (61) | 100 (71) |  |
| Education ^b^, n (%) | | | | | | |
| Elementary education | 5 (12) | 39 (30) | .06 | 5 (16) | 31 (22) | .30 |
| Secondary education | 18 (43) | 46 (36) |  | 8 (27) | 52 (37) |  |
| Higher education | 19 (45) | 44 (34) |  | 17 (57) | 58 (41) |  |
| Marital Status, n (%) | | | | | | |
| Unmarried | 6 (14) | 17 (13) | .83 | 6 (19) | 14 (10) | .14 |
| Married/Partner | 36 (86) | 114 (87) |  | 25 (81) | 127 (90) |  |
| Living arrangements, n (%) | | | | | | |
| Living alone | 4 (10) | 14 (11) | .70 | 7 (23) | 11 (8) | .11 |
| Spouse/Common law partner | 37 (88) | 114 (87) |  | 23 (74) | 124 (88) |  |
| Children | 0 (0) | 2 (1) |  | 1 (3) | 5 (3) |  |
| Other | 1 (2) | 1 (1) |  | 0 (0) | 1 (1) |  |
| EQ-5D-3L index scores ^a,c^ | 0.899 (0.07) | 0.898 (0.08) | .94 | 0.891 (0.08) | 0.889 (0.10) | .91 |
| QoL-AD ^a, c^ | 38.45 (5.90) | 39.71 (5.95) | .23 | 37.19 (6.66) | 39.60 (5.68) | .04 |
| ZBI^a, c^ | 42.95 (5.57) | 43.71 (6.30) | .49 | 40.29 (9.02) | 43.76 (5.80) | .008 |
| Abbreviations: QoL-AD: quality of life in Alzheimer disease; ZBI: Zarit Caregiver Burden Inventory; n number; % percentage  Note*:* Independent sample t-test is used to assess the statistical differences between dropouts and non-dropouts (inter-group [between groups] analysis).  a: Mean (standard deviation); b: n= 129 for non-dropout in the intervention group; n=30 for dropout in the control group; c: n=140 for non-dropout in the control group. | | | | | | |

| **supplementary Table 5**  *Number of healthcare visits and related cost (€) for the participants (mean and standard deviation) at 18-month follow-up --BTH* | | | | | | |
| --- | --- | --- | --- | --- | --- | --- |
|  | PwMCI | | | Informal Caregiver | | |
|  | Intervention (128) | Control  (143) | Difference  (*P* value) | Intervention  (123) | Control  (135) | Difference (*P* value) |
| Outpatient care visits | 19.74 (21.75) | 19.83 (18.80) | 0.09 (.97) | 14.12 (18.03) | 13.33 (15.44) | 0.79 (.71) |
| Inpatient admissions | 0.23 (0.51) | 0.27 (0.61) | 0.04 (.50) | 0.27 (0.67) | 0.22 (0.65) | 0.05 (.58) |
| Inpatient days | 1.11 (3.61) | 1.17 (3.50) | 0.06 (.88) | 1.11 (3.56) | 0.95 (3.43) | 0.16 (.72) |
| Outpatient care cost | 5466 (6947) | 5639 (5777) | -172 (.82) | 3715 (4973) | 3727 (4818) | -12 (.98) |
| Inpatient care cost | 1566 (4123) | 1385 (3292) | 181 (.69) | 1656 (4932) | 1176 (3661) | 480 (.37) |
| Total cost without intervention cost | 7032 (9271) | 7024 (7976) | 9 (.99) | 5371 (8509) | 4903 (7014) | 468 (.63) |
| Total cost including intervention cost | 7206 (889) | 7024 (7976) | 182 (.86) |  |  |  |
| Note*:* Independent sample t-test is used to assess the statistical differences between intervention and control group (inter-group [between groups] analysis). | | | | | | |

| Supplementary Table **6**  *Baseline to 18-month change in health effects within (intra-group differences) intervention and control group and change in health effects between (inter-group differences) intervention and control groups --BTH* | | | | | | | | | | |
| --- | --- | --- | --- | --- | --- | --- | --- | --- | --- | --- |
|  | | Intervention | | | Control | | | Inter-group difference | | |
|  | Sample size^b^ (I/C) | 18-month follow-up | Baseline | Intra-group difference (*P* value) | 18-month follow-up | Baseline | Intra-group difference (*P* value) | Intervention | Control | Difference (*P* value) |
| PwMCI | | | | | | | | | | |
| EQ-5D-3L index score^a^ | 130/141 | 0.887 (0.09) | 0.901 (0.08) | -0.015 (.08) | 0.884 (0.10) | 0.894 (0.09) | -0.010 (.19) | -0.015 (0.09) | -0.010 (0.09) | -0.005 (.72) |
| Composite QoL-AD^a^ | 120/133 | 38.903 (4.83) | 40.428 (4.86) | -1.525 (<.001) | 38. 817 (4.77) | 40.504 (4.83) | -1.687 (<.001) | -1.525 (3.56) | -1.687 (3.82) | 0.162 (.73) |
| MMSE score^a^ | 130/141 | 27.700 (2.68) | 26.654 (1.613) | 1.046 (<.001) | 27.454 (2.80) | 27.000 (1.45) | 0.454 (.02) | 1.046 (2.37) | 0.454 (2.20) | 0.592 (.03) |
| Informal Caregiver | | | | | | | | | | |
| EQ-5D-3L index score^a^ | 127/136 | 0.890 (0.09) | 0.899 (0.08) | -0.009 (.17) | 0.860 (0.12) | 0.888 (0.10) | -0.028 (<.001) | -0.009 (0.08) | -0.028 (0.09) | 0.019 (.07) |
| ZBI^a^ | 126/136 | 44.040 (5.90) | 43.754 (6.40) | 0.286 (.52) | 43.213 (5.72) | 43.691 (5.861) | -0.478 (.32) | 0.286 (4.94) | -0.478 (5.63) | 0.764 (.25) |
| Dyads (PwMCI plus Informal Caregiver) | | | | | | | | | | |
| EQ-5D-3L index score^a^ | 126/136 | 1.780 (0.13) | 1.800 (0.12) | -0.020 (.07) | 1.741 (0.15) | 1.781 (0.13) | -0.039 (.001) | -0.020 (0.12) | -0.039 (0.14) | 0.019 (.25) |
| Abbreviations: MMSE: mini-mental state examination; PwMCI: person with mild cognitive impairment; QoL-AD: quality of life in Alzheimer disease; ZBI: Zarit Caregiver Burden Inventory. a: Mean (standard deviation); bThe number of participants available in the intervention group (I) is followed by the number of participants available in the control group (C).  Note*:* Independent t-test is used to assess the statistical differences between intervention and control group (inter-group [between groups] analysis). Paired t-test is used to assess the intra-group (within intervention and control groups) statistical differences. | | | | | | | | | | |

|  | | | | | | | |
| --- | --- | --- | --- | --- | --- | --- | --- |
|  |  |  |  |  |  |  |  |
|  |  |  |  |  |  |  |  |
|  |  |  |  |  |  |  |  |
|  |  |  |  |  |  |  |  |
|  |  |  |  |  |  |  |  |
|  |  |  |  |  |  |  |  |
|  |  |  |  |  |  |  |  |
|  |  |  |  |  |  |  |  |
|  |  |  |  |  |  |  |  |
|  |  |  |  |  |  |  |  |
|  |  |  |  |  |  |  |  |
|  |  |  |  |  |  |  |  |
|  |  |  |  |  |  |  |  |
|  |  |  |  |  |  |  |  |
|  |  |  |  |  |  |  |  |
|  |  |  |  |  |  |  |  |
|  |  |  |  |  |  |  |  |
|  |  |  |  |  |  |  |  |
|  |  |  |  |  |  |  |  |
|  |  |  |  |  |  |  |  |
|  |  |  |  |  |  |  |  |
|  |  |  |  |  |  |  |  |
|  |  |  |  |  |  |  |  |
|  |  |  |  |  |  |  |  |
|  |  |  |  |  |  |  |  |
|  |  |  |  |  |  |  |  |
|  |  |  |  |  |  |  |  |
|  |  |  |  |  |  |  |  |
|  |  |  |  |  |  |  |  |
|  |  |  |  |  |  |  |  |
|  |  |  |  |  |  |  |  |
|  |  |  |  |  |  |  |  |
|  |  |  |  |  |  |  |  |
|  |  |  |  |  |  |  |  |
|  |  |  |  |  |  |  |  |
|  |  |  |  |  |  |  |  |
|  |  |  |  |  |  |  |  |


| **Supplementary Table 7**  *Inter-group differences of PwMCI --CST* | | | | | | |
| --- | --- | --- | --- | --- | --- | --- |
|  | Baseline | | | 18-month follow-up | | |
|  | Intervention (n=174) | Control (n=173) | *P* value | Intervention (n=74) | Control (n=82) | *P* value |
| Age^a^, years | 74 (8.71) | 74 (7.92) | .63 | 73 (7.72) | 74 (7.61) | .25 |
| Gender, n (%) | | | | | |  |
| Male | 68 (39) | 81 (47) | .15 | 32 (43) | 38 (48) | .60 |
| Female | 106 (61) | 92 (53) |  | 42 (57) | 42 (52) |  |
| Education, n (%) | | | | | |  |
| Elementary education | 125 (72) | 123 (71) | .64 | 55 (74) | 63 (79) | .78 |
| Secondary education | 31 (18) | 27 (16) |  | 12 (16) | 10 (12) |  |
| Higher education | 18 (10) | 23 (13) |  | 7 (10) | 7 (9) |  |
| Marital Status, n (%) | | | | | |  |
| Unmarried | 63 (36) | 56 (32) | .45 | 23 (31) | 22 (28) | .63 |
| Married/Partner | 111 (64) | 117 (68) |  | 51 (69) | 58 (72) |  |
| Living arrangements, n (%) | | | | | |  |
| Living alone | 27 (16) | 29 (17) | .52 | 10 (13) | 9 (11) | .56 |
| Spouse/Common law partner | 95 (56) | 104 (61) |  | 48 (65) | 53 (67) |  |
| Children | 22 (13) | 14 (8) |  | 5 (7) | 2 (3) |  |
| Other | 25 (15) | 24 (14) |  | 11 (15) | 15 (19) |  |
| Composite QoL-AD ^a, b^ | 33.86 (4.25) | 33.06 (4.75) | .10 | 34.25 (4.37) | 33.43 (4.02) | .24 |
| EQ-5D-3L index scores ^a, c^ (Spanish tariff) | 0.764 (0.27) | 0.730 (0.33) | .29 | 0.714 (0.29) | 0.681 (0.31) | .51 |
| MMSE score ^a, d^ | 23.81 (2.63) | 24.38 (2.52) | .04 | 23.40 (5.40) | 23.69 (4.81) | .73 |
| Abbreviations: MMSE mini-mental state examination; PwMCI: person with mild cognitive impairment; QoL-AD: quality of life in Alzheimer disease; n number; % percentage.  Note*:* Independent sample t-test is used to assess the statistical differences between dropouts and non-dropouts (inter-group [between groups] analysis). a: Mean (standard deviation); b: n=172 in the intervention and control groups at baseline; n= 68 in the intervention group & n=75 in the control group at 18-month follow-up; c: n=173 in the intervention group at baseline; n=79 in the control group at 18-month follow-up; d: n=73 in the intervention group & n=80 in the control group at 18-month follow-up. | | | | | | |
